# Are healthcare workers’ intentions to vaccinate related to their knowledge, beliefs and attitudes? a systematic review

**DOI:** 10.1186/1471-2458-13-154

**Published:** 2013-02-19

**Authors:** Raúl Herzog, Mª José Álvarez-Pasquin, Camino Díaz, José Luis Del Barrio, José Manuel Estrada, Ángel Gil

**Affiliations:** 1 Primary Healthcare Service, Madrid Health Service, Santa Hortensia 14, Madrid, Spain; 2 Spanish Association of Vaccinology, Madrid, Spain; 3 Department of Preventive Medicine, Public Health, Medical Immunology and Microbiology, Rey Juan Carlos University, Avenida de Atenas s/n, Alcorcón, Spain; 4 Virtual Library, Lain Entralgo Agency, Gran Vía 27, Madrid, Spain

**Keywords:** Immunization, Vaccination, Knowledge, Belief, Attitude, Healthcare worker, Coverage, Intentions

## Abstract

**Background:**

The Summit of Independent European Vaccination Experts (SIEVE) recommended in 2007 that efforts be made to improve healthcare workers’ knowledge and beliefs about vaccines, and their attitudes towards them, to increase vaccination coverage. The aim of the study was to compile and analyze the areas of disagreement in the existing evidence about the relationship between healthcare workers’ knowledge, beliefs and attitudes about vaccines and their intentions to vaccinate the populations they serve.

**Methods:**

We conducted a systematic search in four electronic databases for studies published in any of seven different languages between February 1998 and June 2009. We included studies conducted in developed countries that used statistical methods to relate or associate the variables included in our research question. Two independent reviewers verified that the studies met the inclusion criteria, assessed the quality of the studies and extracted their relevant characteristics. The data were descriptively analyzed.

**Results:**

Of the 2354 references identified in the initial search, 15 studies met the inclusion criteria. The diversity in the study designs and in the methods used to measure the variables made it impossible to integrate the results, and each study had to be assessed individually. All the studies found an association in the direction postulated by the SIEVE experts: among healthcare workers, higher awareness, beliefs that are more aligned with scientific evidence and more favorable attitudes toward vaccination were associated with greater intentions to vaccinate. All the studies included were cross-sectional; thus, no causal relationship between the variables was established.

**Conclusion:**

The results suggest that interventions aimed at improving healthcare workers’ knowledge, beliefs and attitudes about vaccines should be encouraged, and their impact on vaccination coverage should be assessed.

## Background

Vaccination against preventable diseases is safe and cost-effective, and it has had an important impact on public health worldwide [1]. Because of universal vaccination, various diseases have been eradicated or substantially reduced in many countries [2]. However, vaccine coverage is still not sufficient to control some diseases, such as the measles, which the World Health Organization pledged in 1997 to eradicate from Western Europe by 2007. Recent years have seen repeated outbreaks in this region [3], including epidemics such as the one that occurred in Germany in 2006 [4].

Studies that investigate why a segment of the population does not accept universal vaccination [5-7] have highlighted reasons such as lack of knowledge, misperceptions and distrust in vaccines [8], combined with a low perceived risk of acquiring the disease because the incidence has declined as a result of vaccination programs [9]. Even HCW (healthcare workers) have low vaccine coverage, as is the case with the influenza vaccine, due to various factors, including knowledge about the disease, knowledge about the vaccine or past beliefs and past attitudes (related to influenza vaccination in previous seasons) [10,11].

Despite the significant impact of the media, health professionals have been identified as the most important source of information on vaccination for the general public. Health professionals are key players in recommending vaccination and encouraging the final decision to be vaccinated [12-17]. Therefore, the willingness of health professionals to recommend immunization is crucial. The Summit of Independent European Vaccination Experts (SIEVE) reported in 2007 [18] that strategies to optimize vaccination coverage in children and adults in Europe should be identified and targeted towards healthcare workers (HCWs). SIEVE emphasized the importance of HCWs’ perceptions about vaccines, their attitudes towards them and the need to improve their knowledge of vaccines and increase access to high-quality information about vaccination.

In this context, we posed the following question: Is there a relationship between HCWs’ knowledge of vaccines, their beliefs and attitudes towards them and their intentions to vaccinate the population they serve? To answer this question, we performed a systematic review of the literature on the subject to compile the existing information and identify the areas of disagreement in the knowledge base.

## Methods

First, we performed a pilot study, analyzing the abstracts of the publications we found in our PubMed search that met the inclusion criteria. This process provided an overview of the main features that should be included in the review and allowed us to prepare the protocol for extracting data.

### Search strategy

In June 2009, a literature search was conducted in the PubMed, EMBASE, CINAHL and CENTRAL databases for articles published in one of seven different languages. To ensure that our search was thorough, the search in each database was conducted using both free and controlled language. For the free-text search, the terms included the following: (vaccination OR immunization) AND (healthcare worker OR complementary therapies) AND (knowledge OR beliefs OR attitudes OR barriers). The controlled language search included the following exploded MeSH terms: "Immunization", "Occupational Groups", "Beliefs", "Culture" and "Attitude". The only filter applied was the date of publication; studies were included if they were published between February 1998 and the present (i.e., June 2009). Additional file 1 shows the complete search strategy used in Medline. In addition, for further material, the references of the retrieved articles were manually searched, and key authors were contacted and asked to identify published and unpublished studies that met the inclusion criteria.

### Article selection

After the preliminary selection of potentially relevant titles, two authors (MJA and RH) independently assessed the study abstracts for inclusion or exclusion based on the established criteria (Additional file 2). Based on the abstracts, a consensus decision was made about reading the full text; the full text was read when there was any doubt. To ensure the fulfillment of the inclusion criteria and the exclusion of studies with data collected before the cut-off point, all the studies published after February 1998 that met the inclusion criteria were initially included and read in their entirety by the investigators. Disagreements in the final selection were resolved by consensus, and in cases of continuing disagreement, through consultation with a third reviewer (CD). The reviewers also recorded and compared their reasons for excluding studies, and a consensus was reached when there were disagreements. To determine the degree of agreement in the selection of abstracts and full papers, the Kappa index of inter-observer agreement was calculated using the tool accessible at the following website: http://faculty.vassar.edu/lowry/kappa.html.

### Data extraction and critical appraisal

Each included study was reviewed independently by two investigators (MJA and RH), and the principal characteristics were extracted using a coding method developed in the pilot study. The risk of bias was evaluated using the Newcastle Ottawa scale for case–control studies and an adapted form of the Newcastle Ottawa cohort scale for cross-sectional studies (Additional file 3). Disagreements were resolved by consensus. Results are shown in Table 1.

**Table 1:** Results of the critical appraisal of the included studies

**Study (first author)**	**Study design**	**Selection**				**Comparability**	**Outcome**		
		**Representativeness of the sample**	**Sample size**	**Non-respondents**	**Ascertainment of exposure**	**Based on design and analysis**	**Assessment of outcome**		**Statistical test**
Gonik et al. (2000) [19]	Cross-sectional	+			+		+		
Schupfner et al. (2002) [20]	Cross-sectional	+	+		+	++	+		+
Taylor et al. (2002) [21]	Cross-sectional				+	++	++		+
Zimmerman et al. (2002) [22]	Cross-sectional	+		+	+	++	+		+
Davis et al. (2003) [23]	Cross-sectional	+		+	+	++	+		+
Milledge et al. (2003) [24]	Cross-sectional	+	+	+	+	++	+		+
Jungbauer-Gans et al. (2003) 1st part [25]	Cross-sectional	+			+		+		+
Jungbauer-Gans et al. (2003) 2nd part [25]	Cross-sectional	+			+		++		+
Wilson et al. (2004) [26]	Cross-sectional	+	+	+	+	++	+		+
Russell et al. (2004) [27]	Cross-sectional	+	+	+	+	++	+		+
Petousis-Harris et al. (2005) [28]	Cross-sectional	+	+		+		+		
Clark et al. (2006) [29]	Cross-sectional	+			+		+		
Davis et al. (2007) [30]	Cross-sectional	+	+		+		+		+
Gust et al. (2008) [31]	Cross-sectional	+			+	++	+		+
Goodyear-Smith et al. (2009) [32]	Cross-sectional	+	+	+	+	++	++		+
**Study (first author)**	**Study design**	**Selection**				**Comparability**	**Exposure**		
		**Case definition adequate?**	**Representativeness of the cases**	**Selection of controls**	**Definition of controls**	**Based on design and analysis**	**Ascertainment of exposure**	**Same method for cases and controls**	**Non-response rate**
Salmon et al. (2008) [33]	Case–control	+	+	+	+	+		+	

The results of the included studies are shown in tables. Three tables show the associations found in the cross-sectional studies between the intention to vaccinate and HCWs’ knowledge (Table 2), beliefs (Table 3) and attitudes (Table 4). A fourth table shows the results of the case–control study (Table 5). All studies that aimed to show a possible relationship or an association between the variables were included; there were no restrictions based on the statistical methods used. The adjustments used in the statistical analysis in each study are listed in the last column of the tables, grouped into the following topics: A) Location, B) Demographic characteristics of the patients, C) Practice organization, D) Characteristics of the HCW involved in the study, E) Vaccination-related characteristics, F) HCW experience with morbidity and mortality associated with vaccine-preventable diseases.

**Table 2:** Associations between HCWs’ knowledge and their intentions to vaccinate in cross-sectional studies

**Authors**	**Setting**	**Study population/response rate**	**Determinant (knowledge)**	**Intention to vaccinate**	**Measure of association**	**Adjustments**
Taylor et al. (2002) [21]	USA	112/? pediatricians	Knowledge in vaccine contraindications	Increase of record linked vaccine coverage per each contraindication less stated	At 8 months	2B,1C,3E
					2.0% (95% CI 0.3-3.7) p < 0.05	
					At 19 months	
					2.6% (95% CI 1.1 - 4.7) p < 0.05	
Petousis-Harris et al. (2005) [28]	New Zealand	150/89,3% family practice nurses	Knowledge in vaccine contraindications	Report of vaccination coverage	Significantly greater rate of correct responses in those reporting high coverage (>95) than in those reporting low coverage (<70%). p < 0.05	None
Goodyear-Smith et al. (2009) [32]	New Zealand	106/58% general practitioners	Knowledge in vaccine contraindications	Record linked vaccine coverage	Results shown by region and practice governance:	1A, 2B, 1C, 1D
					Auckland: Maori with right response, median coverage (MC) 30%. Maori with missed response, no practice with these characteristics. Non-Maori right, MC 71%, Non-Maori missed, MC 64%.	
					Midland: Maori right, MC 58%. Maori missed, MC 56%. Non-Maori right, MC 78%. Non-Maori missed, MC 73%.	
					After multivariate analysis, the knowledge remained associated with the coverage (p < 0.05).	

**Table 3:** Associations between HCWs’ beliefs and their intentions to vaccinate in cross-sectional studies

**Authors**	**Setting**	**Study population/response rate**	**Determinant (belief)**	**Intention to vaccinate**	**Measure of association**	**Adjustments**
Zimmerman et al. (2002) [22]	USA	281/72,4% general practitioners, family practice, pediatricians	Perception of: A) Efficacy of the vaccine. yes vs no	Would recommend vaccination	A) To children 12–18 months: 85% vs 70% (p < 0.05)	1A, 1C, 1D, 3E, 1F
					To children 4–6 years: 85% vs 80% (p < 0.05)	
					To children 11–12 years: 86% vs 83% (p > 0.05)	
			B) Storing the vaccine being a major problem. Yes vs no		B) To children 12-18m: 62% vs 86% (p < 0.05) To Children 4–6 years: 73% vs 85% (p > 0.05) To children 11–12 years: 76% vs 87% (p > 0.05)	
Schupfner et al. (2002) [20]	Germany	97/73% pediatricians	Belief that: A) Official vaccination recommendations are influenced by the industry	Reported vaccine coverage rate	A) 60% of those reporting high coverage rate (>80%). 46% of those with low coverage rate (<80%). p > 0.05	2C, 4D, 4E
			B) Behavior in vaccination is mostly conditioned by physician's beliefs		B) 66% of those with high coverage and 59% of those with low coverage. p > 0.05	
Davis et al. (2003) [23]	USA	694/60% family physicians	Believe the new 7-valent pneumococcal vaccine will effectively prevent meningitis	Reported habit of recommending the vaccine	OR 1.86 (95% CI 0.93, 3.73) p > 0.05	4E, 1F
Milledge et al. (2003) [24]	Australia	160/67% general practitioners	Agreement that the following are a deterrent to vaccination: A) Cost- to- parent	Would recommend universal varicella vaccination	A) OR 1.54 (95% CI:0.70-3.38) p > 0.05	1B, 1C, 7E, 2F
			B) Another needle		B) OR 0.79 (0.33–1.49) p > 0.05	
Jungbauer-Gans et al. (2003) [25]	Germany	136/71% family physicians and pediatricians	Training in alternative medicine	Reported habit of recommending full vaccination	With training 63%, without training 78%. p > 0.05 for the difference	None
		94/71% family physicians and pediatricians	Training in alternative medicine	Record linked vaccine coverage	Beta: -0,121 (p < 0.05)	None
Wilson et al. (2004) [26]	Canada	312/59,4% naturopathic students	Belief that: A) Vaccines are beneficial	Willingness to advise full vaccination	A) OR: 16.4 (95% CI 5.15–73.6) p < 0.05	1D, 5E
			B) Vaccines are risky		B) OR: 0.30 ( 0.11–0.74) p < 0.05	
Russell et al. (2004) [27]	Canada	503/78,2% chiropractors	Belief that: A) Vaccines are safe and efficacious	Reported habit of recommending vaccination	A) OR 25.2 [95% CI 8.7-72.7] p < 0.05	2D, 4E
			B) Social orientation of heath		B) OR 2.9 [95% CI 1.7-5.1] p < 0.05	
			C) Broad view of chiropractic practice		C) OR 0.6 [95% CI 0.3-1.1] p > 0.05	
			D) People are informed		D) OR 1.5 [IC95 % 0.9–2.5] p > 0.05	
			E) Chiropractors should recommend vaccination		E) OR 0.9 [IC95 % 0.5–1.4] p > 0.05	
			F) I believe in physicians who think I should recommend vaccination		F) OR 1.5 [IC95 % 1.0–2.4] p > 0.05	

**Table 4:** Associations between HCWs’ attitudes and their intentions to vaccinate in cross-sectional studies

**Authors**	**Setting**	**Study population/response rate**	**Determinant (attitude)**	**Intention to vaccinate**	**Measure of association**	**Adjustments**
Gonik et al. (2000) [19]	USA	313/43% Obstetrician-gynecologists	Assess routinely the patients for vaccine- preventable diseases	Reported habit to administer vaccines	Spearman rho correlation 0.30–0.70; p < 0.05	None
Zimmerman et al. (2002) [22]	USA	281/72,4% general practitioners, family practice, pediatricians	Agreement with the national recommendations on varicella vaccination. Yes vs no	Would recommend the vaccination	In children 12–18 months: 98%	1A,1C, 1D,3E, 1F
					vs 3%, p < 0.05	
					Children 4–6 years: 93%	
					vs 19%, p < 0.05	
					Children 11–12 years:	
					86% vs 68%, p < 0.05	
Taylor et al. (2002) [21]	USA	112/? pediatricians	A) Number of injections willing to give in one visit. Range 1 to 6 (>5)	Increase of record linked vaccine coverage	A) Per each injection more: Increase at 8 months of 3.6% (95% CI 0.4-6.8) p > 0.05, at 19 months 1.5% (95% CI −2.8 - 5.5) p > 0.05	2B, 1C, 3E
			B) Recommendation of inactivated polio vaccine (IPV) vs oral vaccine		B) Using IPV: Increase at 8 months of 8.9% (95% CI 3.3-15.4) p < 0.05, at 19 months 15.4% (95% CI 7.7 - 23.1) p < 0.05	
Schupfner et al. (2002) [20]	Germany	97/73% pediatricians	Prefer to give combined vaccines than separate	Reported vaccine coverage rate	100% in those with high reported coverage (>80%) vs 81% in low coverage (<80%) p > 0.05	2C,4D,4E
Milledge et al. (2003) [24]	Australia	160/67% general practitioners	Concerns about varicella vaccine: A) Immunity may not be life-long	Would recommend universal varicella vaccination	A) OR 0.60 (95%CI 0.33-1.21) p > 0.05	1B, 1C, 7E, 2F
			B) Increase in herpes zoster		B) OR 1.08 (0.33-3.6) p > 0.05	
			C) More serious varicella disease in adults		C) OR 0.92 (0.37-2.27) p > 0.05	
			D) Possible, unknown side effects		D) OR: 0.31 (0.15–0.63) p > 0.05	
Davis et al. (2003) [23]	USA	694/60% family physicians	A) Considers giving 5 injections at 1 visit vs less	Reported habit of recommending the vaccine	A) OR 17.29 (95% CI 6.35, 47.05) p < 0.05	4E, 1F
			B) Considers giving 4 injections at 1 visit vs less		B) OR 8.69(95% CI 4.21, 17.94) p < 0.05	
Jungbauer-Gans et al. (2003) [25]	Germany	136/71% family physicians and pediatricians	Importance of the officially recommended vaccinations (Index: 1 = not at all, 5 = very)	Reported habit of recommending full vaccination	Index of 4.8 in those recommending full vaccination vs 3.9 in those who did not. p < 0.05	None
		94/71% family physicians and pediatricians	Importance of the officially recommended vaccinations (same Index)	Record linked vaccine coverage	One point increase in the Index was associated with an increase of 25.8% in the coverage. p < 0.05	None
Wilson et al. (2004) [26]	Canada	312/59,4% naturopathic students	Trust in Public Health information	Willingness to advise full vaccination	OR 3.72 (95% CI 1.42–10.7) p < 0.05	1D, 5E
Clark et al. (2006) [29]	USA	183/54% obstetricians	Perceive to have a role in promote Tdap vaccination to other adults (not mothers) in contact with infants	Report to recommend Tdap vaccine to pregnant women	77% perceive having a role in those recommending vaccine to pregnant women vs 50% in those who do not. p < 0.05	None
Davis et al. (2007) [30]	USA	336/49% family physicians, general internists	Agree that pertoussis is serious enough to warrant using Tdap in adults. Yes vs no or neutral	Would recommend the vaccination if recommended	93% vs 68%. p < 0.05	None
Gust et al. (2008) [31]	USA	733/65% family physicians, pediatricians	Have some concerns about immunization	Recommend full immunization	OR 0.32 (95% CI 0.56-0.19) p < 0.05	1C, 1D, 1E

**Table 5:** Associations between HCWs’ beliefs and attitudes and their intentions to vaccinate in case–control studies

**Authors**	**Setting**	**Study population/respnse rate**	**Determinant**	**Intention to vaccinate**	**Measure of association**	**Adjustments**
Salmon et al. (2008) [33]	USA	Sample size 551 (55 cases/432 controls/64 mixed ^c^ ). Primary healthcare professionals	**Beliefs**	Cases: Primary healthcare professionals of unvaccinated		Medical doctors or doctors in osteopathy
			A1) Disease susceptibility		A1) OR 1.39 (95%CI:0.68–2.85) p < 0.05	
			A2) Disease severity	children at school entry. vs Controls: Primary healthcare professionals only of vaccinated children.	A2) OR 0.90 (0.59–1.38) p < 0.05	
			A3) Vaccine efficacy		A3) OR 1.37(0.65–2.86) p < 0.05	
			A4) Vaccine security		A4) OR 0.37 (0.19-0.72) p < 0.05	
			B) Benefit when a child is fully vaccinated for: B1) Child		B1) OR 0.30 (0.10–0.85) p < 0.05	
			B2) Community		B2) OR 0.28 (0.09–0.88) p < 0.05	
			B3) Primary care practitioner		B3) OR 0.59 (0.39–0.90) p < 0.05	
			B4) Insurance company		B4) OR 0.56 (0.32–0.99) p < 0.05	
			B5) Government		B5) OR 0.55 (0.32–0.96) p < 0.05	
			B6) Vaccine companies		B6) OR 0.57 (0.30–1.10) p < 0.05	
			C) Agree or completely agree with the following statements: C1) Children get more immunizations than are good for them		C1) OR 2.28 (1.56-5.10) p < 0.05	
			C2) A good diet is more important		C2)OR 3.68 (1.61-8.38) p < 0.05	
			C3) Child’s immune system could be weakened		C3) OR 4.03 (2.06-7.86) p < 0.05	
			C4) Better to develop immunity by getting sick		C4) OR 4.08 (1.9-8.76) p < 0.05	
			**Attitudes**			
			A) Should be allowed to send unvaccinated children to school		A) 1.72 (1.13-2.6) p < 0.05	
			B) Worry that many of the reports of serious side effects from vaccines are accurate		B) 2.03 (1.05-3,91) p < 0.05	
			C) Concerned the CDC/ACIP underestimates the frequency of vaccine side effects		C) 2.86 (1.65-4.97) p < 0.05	

^C^Mixed providers were those who had vaccinated and unvaccinated children. A binomial framework allowed for mixed providers to contribute to the odds ratio calculation. CDC: Centres for Disease Control and Prevention. ACIP: Advisory Commitee on Immunization Practices.

To evaluate whether the methodological quality of the included studies influenced the direction or the magnitude of the results, we performed a separate analysis on those that complied with stricter inclusion criteria: 1) using vaccination coverage (measured by vaccination registries) as a measurement of the intention to vaccinate and 2) controlling for the main confounding factors in the statistical analysis. In these studies, it is less likely that the intention to vaccinate was overestimated, compared to the studies that rely on personal evaluation. In addition, the association between the variables is less susceptible to bias caused by confounding factors; thus, the internal validity is greater. Using these filters, we performed a sensitivity analysis. We measured the changes that were observed in the associations when only the studies with greater internal validity were included, compared to the results when we included all the studies that met the initial criteria.

Based on the consensus between two authors (MJA and RH), the different questions posed to the HCW in the studies were grouped by topic. Table 6 shows a summary of the associations found between each topic or factor and HCWs’ intentions to vaccinate. The results of a study were classified as “null association” if the confidence interval included the value representing a null association, if the results were not statistically significant, if confounders were not controlled or if there was insufficient information to interpret the results. To allow for interpretation, some odds ratios had to be inverted to clarify the meaning of the association between the intention to vaccinate and the factors that were evaluated in several studies.

**Table 6:** Summary of factors related to knowledge, beliefs and attitudes and their associations with HCWs’ intentions to vaccinate

**Factor**	**Negative associations**			**Null associations**			**Positive associations**		
	**Logistic regression**		**Other analysis**	**Logistic regression**		**Other analysis**	**Logistic regression**		**Other analysis**
	**N**	**Range OR**	**N**	**N**	**Range OR**	**N**	**N**	**Range OR**	**N**
**1. Knowledge**									
a. Vaccine contraindications.	0		0	0		1	0		2
**2. Beliefs**									
a. Vaccines are more risky than beneficial.	3	0,04-0,37*	0	0		0	0		0
b. Vaccine low efficacy and benefit and low susceptibility and severity of the disease.	2	0,04-0,06	1	2	0,54-1,39*	0	0		0
c. Use of alternative medicine theories	1	0,24*-0,44*	1	1	0,6	0	0		0
d. More individualist than social orientation of the health care.	2	0,28*-0,59*	0	0		0	0		0
e. Cost-to-parent is a deterrent to vaccination.	0		0	1	1,54	0	0		0
f. Another needle is a deterrent to vaccination.	0		0	1	0,79	0	0		0
g. Stocking the vaccine is a problem.	0		1	0		0	0		0
h. People are adequately informed about vaccine.	0		0	1	1,5	0	0		0
i. Theories of conspiration, influence of the farmaceutical industry on the policy makers in immunization.	0		0	0		1	0		0
j. Behavior in vaccination is mostly conditioned by physician's beliefs	0		0	0		1	0		0
k. Chiropractors should counsel about immunization.	0		0	0		1	0		0
**3.Attitudes**									
a. Have some concerns about immunization.	1	0,32	0	0		0	0		0
b. Concerned about vaccine’s side effects.	1	0,49*	0	1	0,31	0	0		0
c. Low confidence in Public Healthcare information or national recommendations.	2	0,27-0,35*	1	0		0	0		0
d. Consider the disease serious enough to warrant using a vaccine or give importance to the vaccination.	0		0	0		0	0		2
e. Number of injections the physician considers giving at one visit (4 vs less and 5 vs less).	0		0	0		1	1	8,69 and 17,29	0
f. Perceive to have a role in vaccination.	0		0	0		1	0		1
g. Preference for combined vaccine than for separate.	0		0	0		1	0		0
h. Concerned about (A) vaccine immunity may not be life-long (B) will lead to more serious disease in adults.	0		0	1	A) 0,6 B)1,08	0	0		0
i. Not having adopted the new recommendations in use of polio vaccine (still using Sabin vs Salk).	0		1	0		0	0		0
j. It should be allowed to send unvaccinated children to school.	1	0,58*	0	0		0	0		0

N: Number of studies OR: Odds Ratio * Results from the case-control study.

Table 7 shows the main characteristics of the studies that were ultimately included, as well as the Kappa index of agreement.

**Table 7:** Characteristics of the included studies

**Authors**	**Knowledge, beliefs, attitudes**	**Measure of intention to vaccinate**^**A**^	**Design**	**Type of tool**	**Mean to collect data**	**Anonymity**	**Questionnaire: new, previously used, validated**	**Target population**^**B**^	**Vaccine studied**^**C**^
Clark et al. [29]	attitude	2	cross-sectional	survey	mail	not specified	new	Obst./Gyn.	DTP
Petousis-Harris et al. [28]	knowledge	4	cross-sectional	survey	telephone	not anonymous	new	Nurses	P,MMR
Wilson et al. [26]	beliefs and attitude	2	cross-sectional	survey	working place or similar	not specified	new	Nat. stu.	PedV
Jungbauer-Gans et al. [25]	beliefs and attitude	1 and 3	cross-sectional	survey and record linked	not specified and record linked	not specified	not specified	FP/GP, Ped.	PedV
Milledge et al. [24]	beliefs and attitude	2	cross-sectional	survey	mail	not specified	not specified	FP/GP	Var.
Zimmerman et al. [22]	beliefs and attitude	2	cross-sectional	survey	mail, e-mail or web, telephone	not anonymous	new	FP/GP, Ped.	Var.
Davis et al. [30]	attitude	2	cross-sectional	survey	mail	not specified	not specified	FP/GP, Int.	DTP
Davis et al. [23]	beliefs and attitude	1	cross-sectional	survey	mail	not specified	new	FP/GP, Ped.	Pn.
Russell et al. [27]	beliefs	1	cross-sectional	survey	mail	not specified	new	Chiropractors	GenV
Taylor et al. [21]	knowledge and attitude	3	cross-sectional	survey and record linked	working place and record linked	not anonymous	not specified	Ped.	DTP,MMR, Hep. B,HiB,Pol.
Gust et al. [31]	attitude	1	cross-sectional	survey and record linked	e-mail or web	anonymous	previously used	FP/GP, Ped.	PedV
Gonik et al. [19]	attitude	1	cross-sectional	survey	mail	anonymous	new	Obst./Gyn.	DT, MMR, Hep.B, Var.
Schupfner et al. [20]	beliefs and attitude	4	cross-sectional	survey	mail	anonymous	new	Ped.	PedV
Goodyear-Smith et al. [32]	knowledge	3	cross-sectional	survey and record linked	telephone and record linked	not anonymous	previously used, modified	FP/GP	PedV
Salmon et al. [33]	beliefs and attitude	1	case and control	survey and record linked	mail	not anonymous	not specified	PHCP, DO	SchV
**Kappa index**	0,65	0,71	1	0,72	0,9	0,89	0,9	0,92	0,83

A) 1) Reported habit of vaccination or recommending vaccination to the patients. 2) Reported intention to vaccinate or recommend vaccination to the patients. 3) Record linked vaccination coverage. 4) Reported vaccination coverage. B) FP/GP: family practice and general practice. Ped.: pediatrician. Int.: internist. Obst./Gyn.: obstetrician and gynecologist. Nat. stu.: naturopathy students. PHCP: primary healthcare provider. DO: doctors in osteopathy C) DT: difteria, tetanus. P: pertussis. MMR: measles, mumps, rubella. Hep.B: B hepatitis. HiB: *Haemophilus influenzae* type B. Pol.: polio Var: varicella. Pn.: antipneumoccocal vaccine for children. GenV: vaccine in general. PedV: vaccines recommended in pediatrics. SchV: vaccine required for school entry.

## Results

**Figure 1:**
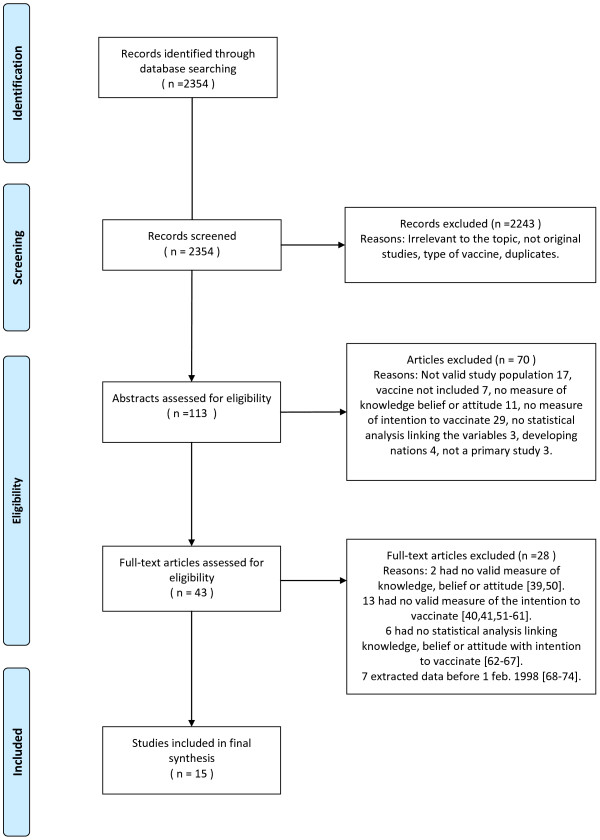
**Flow chart of the reviewing process **[39-41,50-74]**.**

Of the 2354 references identified in the initial search, 113 were pre-selected because they aligned with the study objectives. After applying the inclusion criteria to the abstracts, the full-text reports of 43 studies were evaluated. Of these, 15 met the inclusion criteria and were included in the final analysis. A flow chart illustrating the studies that were excluded at each stage in the review process is shown in Figure 1.

The Kappa index was 0.653 for the selection of the full-text studies to be read after applying the inclusion criteria to the abstracts, and it was 0.778 for the final inclusion of the studies. Of the studies ultimately selected, 14 were cross-sectional studies [19-32], and one was a case–control study [33]. We found no other published systematic reviews on this topic.

Three studies analyzed the relationship between the knowledge of HCW and their intentions to vaccinate, eight studied the relationship between beliefs and intentions to vaccinate, and 12 studied the relationship between attitudes and intentions to vaccinate. Seven studies analyzed the relationships between two of these variables and intentions to vaccinate.

The critical appraisal of the included studies is shown in Table 1.

### HCWs’ knowledge of vaccines and their intentions to vaccinate

Three cross-sectional studies analyzed the relationship between HCWs’ knowledge and the intention to vaccinate. Their results are shown in Table 2. These studies included 106 to 150 participants, with a mean of 123 participants and a total of 368 participants. Two were performed in New Zealand and one in the USA. The three studies used different methods to measure knowledge, but they all found a significant association between knowledge and the intention to vaccinate, such that the greater the knowledge level, the greater the intention to vaccinate.

Two studies used multivariate analysis to control the influence of confounders and also used record-linked vaccination coverage as a measure of the intention to vaccinate. The results of these two studies are not comparable with the third study, because they used different tools to measure knowledge and intentions to vaccinate, as well as to measure the association. Therefore, subgroup analysis does not allow us to compare the magnitude of the association, but it does show the same direction of the association between the variables, independent of the internal validity of the studies.

### HCWs’ beliefs about vaccines and their intentions to vaccinate

Seven cross-sectional studies and one case–control study collected information on HCWs’ beliefs and their relationship with intentions to vaccinate. The sample size in the eight studies ranged between 94 and 694, with a mean of 341 and a total of 2731 HCW. Three studies were performed in the USA, two in Germany, two in Canada and one in Australia. The results of the cross-sectional studies are shown in Table 3, and those from the case–control study are shown in Table 5.

Only the case–control study by Salmon et al. measured the intention to vaccinate using vaccination records and also controlled for confounding factors. It was not possible to compare the results of this study with the others included, because each study asked specific but different questions about beliefs. In spite of these differences, the sensitivity analysis shows that the study with the greatest internal validity found the same direction of association as the other studies: the beliefs most aligned with scientific evidence were associated with greater intentions to vaccinate among HCW.

Of the eight studies, four studied only conventional HCW (pediatricians, family and general practitioners), and two studied complementary or alternative medicine providers (chiropractors, naturopathy students). The difference between the beliefs held by these two groups could not be evaluated by comparing these studies because of the differences in the variables discussed above. However, the remaining two studies did compare the two types of HCW. One of them (Jungbauer-Gans et al.) compared family practitioners and pediatricians with and without training in naturopathy. They found that the physicians trained in naturopathy reported that they prescribed fewer vaccines (considering all the vaccines recommended by the competent authority in Germany (STIKO)) than the physicians without this training (63% vs. 78%, respectively), although the difference was not statistically significant. Likewise, the physicians trained in naturopathy had a significantly lower proportion of patients with up-to-date vaccinations (Beta: -0,121).

The case–control study (Salmon et al.) included different types of primary healthcare professionals. The study found that the conventional HCW made up a greater proportion of the control group (HCW whose patients were all fully vaccinated upon school entry), than the group of cases (those who were responsible for children with non-medical vaccination exemptions) (87.9% vs. 74.1%, respectively; p < 0.05). The doctors of osteopathy made up a greater proportion of the cases than the controls (13% vs. 5.4%, respectively; p < 0.05). However, the study was not designed to analyze the differences according to provider training, and it did not control for confounders.

### HCWs’ attitudes towards vaccines and their intentions to vaccinate

We found 11 cross-sectional studies and one case–control study that examined the relationship between HCWs’ attitudes and their intentions to vaccinate. The results of the cross-sectional studies are shown in Table 4, and those from the case–control study are shown in Table 5. The sample size of the 12 studies ranged between 94 and 694 HCW, with a mean of 326 and a total of 3908. Eight studies were performed in the USA, two in Germany, one in Canada and one in Australia.

Seven of the 11 cross-sectional studies controlled for confounders, but only one included vaccination records (Taylor et al.). The case–control study (Salmon et al.) had both characteristics. These two studies were included in the sensitivity analysis. Each study included specific but different questions that explored attitudes, and therefore, the results are not comparable. However, two aspects about attitudes were examined by one of the two studies with greater internal validity and one other study. Thus, for the sensitivity analysis, we compare the results of these studies.

Taylor et al. and Davis et al. (2003) investigated the number of injections that HCW were willing to give in the same medical visit. Taylor et al. studied the record linked percentage of children with up-to-date vaccinations, and Davis et al. studied the self-reported habit of administering a recently-recommended injectable vaccine. Both studies found a positive association between the willingness to give a number of injections simultaneously and compliance with the recommended vaccination schedule, although the association did not remain significant after multiple regression analysis in the study by Taylor et al. The sensitivity analysis should take the lack of statistical significance into account, but this approach does not necessarily invalidate the results of Davis et al. In addition to the methodological differences, there were other basic differences, such as the type of vaccine studied and the associations explored.

Salmon et al. and Milledge et al. studied concerns about the known and unknown side effects of vaccination. Milledge et al. found that concerns about the possible unknown side effects of vaccination made HCW less willing to recommend it (OR: 0.31 (95% CI: 0.15-0 .63); p > 0.05). Salmon et al. found that concerns about the accuracy of the reported severe side effects of vaccination were more strongly associated with cases (i.e., physicians who signed non-medical exemptions from vaccination) than controls (OR: 2.03 (95% CI: 1.05-3.91); p <0.05). In both studies, the concern about side effects was associated with lower adherence to vaccination recommendations, and the study with the highest internal validity had statistically significant results. Therefore, the sensitivity analysis does not change the direction of the association.

### Summary of associations

Table 6 shows a summary of the associations found in the studies between specific factors explored as knowledge, beliefs and attitudes, and HCWs’ intention to vaccinate. To measure knowledge, the three included studies evaluated the knowledge in vaccination contraindications. Three topics out of the nine on belief evaluations, showed up in more than two studies: “Vaccine low efficacy and benefit and low susceptibility and severity of the disease” (present in 5 studies), “Vaccine are more risky than beneficial” (3 studies), “Use of alternative medicine theories” (3 studies). Only one topic out of the ten exploring attitudes appeared in more than two studies: “Low confidence in Public Healthcare information or national recommendations”, (3 studies).

### Characteristics of studies included

Table 7 shows the characteristics of each of the studies included in the review and the Kappa index of inter-observer agreement on the classification of the study characteristics.

## Discussion

This review identifies and summarizes the quantitative evidence about the possible relationship between HCWs’ knowledge, beliefs and attitudes about vaccines and their intentions to vaccinate. The results of the included studies clearly show that these relationships do exist, although unfortunately, the data does not allow us to make conclusions about a causal link, mainly because all but one of the studies are cross-sectional. Only one retrospective case–control study could show a causal link between beliefs, attitudes and intentions to vaccinate, but even this study should be considered as cross-sectional because it evaluated HCWs’ beliefs and attitudes at a given point in time, and these variables may change over time.

Given the range of the inclusion criteria, the included studies differed widely in their evaluations of the variables, methodologies, and statistical analysis techniques. Therefore, the results cannot be integrated to quantify the magnitude of the associations and must be evaluated individually. Even so, it appears that all the studies show associations in the direction postulated by the SIEVE experts, although some associations were statistically significant, and others were not.

Knowledge measurement was only based on questions on vaccination contraindications, which may imply that researchers were especially worried about the importance of this factor in regard to the intention to vaccinate. On the other hand, the variety of topics that were explored such as beliefs and attitudes is wide, as is shown in Table 6, though few were repeated in various studies. This may denote interest of the researchers on showing the importance of this type of factors on the intention to vaccinate. In regard to beliefs, the topics that made reference to efficacy and security of the vaccines, severity of the vaccine preventable disease and use of alternative medicine theories, appeared in various studies and seem to be perceived by the researchers as important factors. The associations found with the intention to vaccinate, show this importance. As for attitudes, only “Low confidence in Public Healthcare information or national recommendations” factor was explored in more than two studies (in 3 out of 12 that measured attitudes). This may reflect that there doesn´t exist a clear investigation line intended to prove the importance of a specific factor. It´s also interesting to highlight that the knowledge, beliefs and attitude themes explored in the HCW in the included studies, do not differ from the barriers towards vaccination explored in the general population [6].

The results of this study must be understood in the context of the limitations of the methodology used. Including observational studies in systematic reviews presents specific challenges, as observational studies have inherent biases (mostly selection and information bias) and vary in their study designs. Taking these concerns into account, we have attempted to be as rigorous as possible in the methodology of this review [34].

We tried to reduce identification bias by performing the literature search in four databases in addition to a manual search, selecting studies in seven languages and making personal contact with authors when necessary. We did not search for unpublished articles or for “grey literature”, and there may be unidentified articles in databases we did not search. Consequently, there may be a risk of publication bias. One limitation was that we chose to include only the studies that started collecting data after the publication of the article by Wakefield et al. This article should not imply that changes occurred in the relationship between knowledge and the intention to vaccinate, and therefore, there may have been studies before this date whose results were equally valid for understanding the current situation. On the other hand, the included studies were those published up to June 2009, so at the time of its publishing our study won´t include the latest evidence. In spite of this, having not found other systematic reviews on this topic, the results may be of interest to investigators, policy makers and healthcare professionals. In addition, we only included data from developed countries. In other words, there may have been relevant studies from developing countries that were not included and also that the results cannot be extrapolated to these countries. HCWs’ perceptions of vaccines and of vaccine-preventable diseases may be different in developing countries because they face different disease burdens, and this is the reason why these studies were not included [35,36].

One difficulty we encountered was classifying and distinguishing the information related to knowledge, beliefs and attitudes, despite the definitions we established, as shown by the low Kappa index for inter-observer agreement. It is also likely that this difficulty in delimiting the variables caused the low Kappa index for the abstract selection, and we believe that an intentional training of the researchers in identifying and classifying the variables during the pilot study would have improved the concordance.

The tools used to determine the risk of bias and the quality of the studies included were based on the Newcastle-Ottawa scales, which are widely used for observational studies [37]. The deficiencies in the methodology and reporting of many of the studies are reflected in the low scores on the quality scales, questioning the reliability of the studies reviewed. With regard to the sample selection, almost all of the studies used an acceptable sampling method, but most failed to report the comparability of the respondents and the non-respondents, which may imply there was a self-selection bias. Many included studies used subjective measures (e.g., self-reporting, unverified intentions or behaviors), which can lead to information bias. Care must be taken in interpreting such information, as there is a tendency for respondents to provide what they believe to be socially acceptable answers [38]. Some studies did not control for confounders, such as demographic factors, meaning that the variability in the intentions to vaccinate may be incorrectly associated with knowledge, beliefs or attitudes. The studies also failed to use validated instruments to measure attitudes, knowledge and beliefs. In addition, there are factors related to immunization that fall in these three areas but were not measured in the included studies, so these studies may offer only a partial viewpoint of the relationship between HCWs’ attitudes, knowledge and beliefs and intentions to vaccinate.

This review fills a gap in the literature, and thus, despite the limitations of our methodology, we believe that the benefits of illuminating this relevant topic overcome the limitations. Qualitative studies, which were not considered in this review, could be the objective of a future review. A gold standard research study is a broad-based population study that controls for confounding factors and biases and uses validated tools to measure knowledge, beliefs, attitudes, and intentions to vaccinate or vaccine coverage. The knowledge, beliefs and attitudes that may determine the intention to vaccinate are those stated at the moment when the intent to vaccinate is measured. The fact that these variables change over time may explain the absence of longitudinal studies that are capable of demonstrating causality. Despite this lack of evidence, various studies have evaluated the impact of interventions on the intent to vaccinate or the practice of vaccination [25,39-44], and others have even assessed their cost-effectiveness [45].

Some years ago, it was stated that the success of vaccination programs depends on strong professional commitment, that it is important to have written clinical guidelines to strengthen, instruct and support professionals at the time of vaccination, and that effective use of information technology would be beneficial [46]. On the last two points, great strides have been made; it may be time, as the SIEVE experts state, to test and implement strategies to incentivize health professionals.

## Conclusions

The available information shows that among HCW, greater knowledge about vaccines, beliefs that are aligned with scientific evidence and more favorable attitudes toward vaccines are associated with greater intentions to vaccinate. However, it is not possible to conclude that there is a causal relationship between these variables, because the included studies are observational and must be interpreted as cross-sectional. The fact that knowledge, beliefs and attitudes change over time may explain the absence of longitudinal studies capable of demonstrating causality. We conclude that the existing studies show associations between HCWs’ knowledge, beliefs and attitudes and their intentions to vaccinate the populations they serve. The next step is to test and implement interventions and strategies focused on the knowledge, beliefs and attitudes of HCW to attempt to improve vaccine coverage.

## Abbreviations

HCW: Healthcare worker; SIEVE: Summit of Independent European Vaccination Experts.

## Competing interests

The authors declare that they have no competing interests.

The Spanish Vaccinology Foundation financed the translation of the article.

## Authors’ contributions

RH participated in the study design, the article search, the study selection process, the data extraction and analysis, and the drafting of the manuscript. MJA conceived the study and participated in the study design, the study selection process, the data extraction and analysis, and the drafting of the manuscript. CD assisted with the study selection process and the drafting of the manuscript. JLB assisted with the methodological aspects of the systematic review and the statistical interpretation of the included studies and revised the manuscript. JME performed the search in the electronic databases. AG participated in the design and coordination of the review, revised the manuscript and gave his approval for the manuscript to be published. All authors read and approved the final manuscript.

## Pre-publication history

The pre-publication history for this paper can be accessed here:

http://www.biomedcentral.com/1471-2458/13/154/prepub

## Supplementary Material

Additional file 1 Search strategy for Medline. Click here for file

Additional file 2 **Inclusion and exclusion criteria.** Inclusion and exclusion criteria for the selection of studies in this review [35,36,47-49]. Click here for file

Additional file 3 **Critical appraisal tool for cross-sectional studies.** Modified from the Newcastle-Ottawa Quality Assessment Scale for Cohort Studies. Click here for file
