# Supplementary material for: Are healthcare workers’ intentions to vaccinate related to their knowledge, beliefs and attitudes? a systematic review
Source: BMC Public Health. 2013 Feb 19;13:154. doi: 10.1186/1471-2458-13-154 (PMC3602084; doi:10.1186/1471-2458-13-154)
Supplement: Additional file 1 — Search strategy for Medline. [file 1471-2458-13-154-S1.docx]

Search strategy for Medline using OVID.

| # | 32 |  | #30 AND #31 |
| --- | --- | --- | --- |
| # | 31 |  | ('knowledge'/exp OR 'knowledge') OR attitude* OR ('culture'/exp OR 'culture') AND [1998-2009]/py |
| # | 30 |  | #22 AND #29 |
| # | 29 |  | #18 OR #28 |
| # | 28 |  | #23 OR #24 OR #25 OR #26 OR #27 |
| # | 27 |  | ('alternative therapies'/exp OR 'alternative therapies') OR ('alternative therapy'/exp OR 'alternative therapy') OR ('anthroposophy'/exp OR 'anthroposophy') OR ('complementary medicine'/exp OR 'complementary medicine') OR ('complementary therapies'/exp OR 'complementary therapies') OR ('diet fads'/exp OR 'diet fads') OR ('laughter therapy'/exp OR 'laughter therapy') AND or; AND ('medicine alternative'/exp OR 'medicine alternative') OR ('mental healing'/exp OR 'mental healing') OR ('mind body technique'/exp OR 'mind body technique') OR ('mind body therapies'/exp OR 'mind body therapies') OR ('mind body therapy'/exp OR 'mind body therapy') OR 'mind-body relations' OR ('naturopathy'/exp OR 'naturopathy') OR ('orthomolecular medicine'/exp OR 'orthomolecular medicine') OR ('polarity therapy'/exp OR 'polarity therapy') OR ('radiesthesia'/exp OR 'radiesthesia') OR ('reflexotherapy'/exp OR 'reflexotherapy') OR ('shamanism'/exp OR 'shamanism') OR ('spiritual therapies'/exp OR 'spiritual therapies') OR ('therapeutic cults'/exp OR 'therapeutic cults') OR ('therapeutic touch'/exp OR 'therapeutic touch') AND [1998-2009]/py |
| # | 26 |  | ('complementary therapies'/exp OR 'complementary therapies') AND [1998-2009]/py |
| # | 25 |  | ('alternative therapies'/exp OR 'alternative therapies') AND [1998-2009]/py |
| # | 24 |  | ('complementary medicine'/exp OR 'complementary medicine') AND [1998-2009]/py |
| # | 23 |  | ('alternative medicine'/exp OR 'alternative medicine') AND [1998-2009]/py |
| # | 22 |  | #19 OR #20 OR #21 |
| # | 21 |  | nurse* AND [1998-2009]/py |
| # | 20 |  | physician* AND [1998-2009]/py |
| # | 19 |  | ('health personnel'/exp OR 'health personnel') AND [1998-2009]/py |
| # | 18 |  | #11 OR #12 OR #13 OR #14 OR #15 OR #16 OR #17 |
| # | 17 |  | ('vaccination'/exp OR 'vaccination') AND [1998-2009]/py |
| # | 16 |  | ('immunization passive'/exp OR 'immunization passive') AND [1998-2009]/py |
| # | 15 |  | ('mass immunization'/exp OR 'mass immunization') AND [1998-2009]/py |
| # | 14 |  | ('immunization schedule'/exp OR 'immunization schedule') AND [1998-2009]/py |
| # | 13 |  | ('immunization secondary'/exp OR 'immunization secondary') AND [1998-2009]/py |
| # | 12 |  | ('immunization programs'/exp OR 'immunization programs') AND [1998-2009]/py |
| # | 11 |  | ('immunization'/exp OR 'immunization') AND [1998-2009]/py |
