# Supplementary material for: Are healthcare workers’ intentions to vaccinate related to their knowledge, beliefs and attitudes? a systematic review
Source: BMC Public Health. 2013 Feb 19;13:154. doi: 10.1186/1471-2458-13-154 (PMC3602084; doi:10.1186/1471-2458-13-154)
Supplement: Additional file 2 — Inclusion and exclusion criteria. Inclusion and exclusion criteria for the selection of studies in this review [35,36,47-49]. [file 1471-2458-13-154-S2.docx]

1.     Study population:

The target study population should be HCW who administer or recommend vaccines to the population or to specific population groups and have direct contact with patients.

HCW: Physicians and nurses from any specialty, dentists, pharmacists, chiropractors, homeopaths, acupuncturists, any complementary or alternative medicine providers, students or residents of any of the aforementioned groups and any professional group involved in administering or recommending vaccines in their community.

2.     Knowledge, beliefs and attitudes:

The study should collect data on at least one of the following three characteristics: knowledge, beliefs or attitudes of the target population related to vaccination, defined as:

Knowledge: Information possessed by the health worker about vaccines, evaluated by asking questions about scientific evidence, protocols or other issues related to vaccines and their indications.

Beliefs: Thoughts that are not based on facts. Thoughts without a rational basis, or based on information that is not aligned with scientific evidence, or with no knowledge of a rational basis. As Rivera says [47], “we count on beliefs and thus, we do not think about them consciously”. We artificially divided conventional providers and complementary or alternative medicine providers into two belief groups*.*

Attitudes: A posture or opinion about vaccines or vaccination that involves a vaccine-related act or its omission. A positive or negative disposition towards vaccination and related activities.  According to Rivera, attitudes, which ultimately translate into behavior, are based on a myriad of unconscious beliefs and expectations.

It is sometimes difficult to distinguish between beliefs and knowledge. When in doubt, we considered the question posed. If it was presented as something that the subject should know, it was considered to be knowledge-related. It was considered to be belief-related if the question used phrases such as, "Do you believe…?" or "Do you think…?"

3. Intentions to vaccinate:

The study should include some measure of HCWs’ intentions to vaccinate the populations they serve. This goal can be achieved by asking direct questions about the intention to vaccinate or practice of vaccinating the population served by the HCW, or by objective evidence of HCW vaccination practices (e.g., vaccination registry, patient questionnaire, etc.), as the intention to vaccinate is a surrogate for vaccination itself. We also included questions about intentions to recommend vaccination and the practice of recommending vaccination to users.

4. Type of study:

Original, analytical studies that use statistical methods to link the variables "knowledge, beliefs and attitudes" with the variable "intention to vaccinate."

5. Type of vaccines studied:

The study should be about immunization in general, or it should be specifically focused on the following vaccines routinely included in the majority of European vaccination schedules: diphtheria, pertussis, tetanus, polio, measles, mumps, rubella, *Haemophilus influenzae* type B, hepatitis B, varicella, *Streptococcus pneumoniae* in childhood and meningococcal C.

6. Time period:

The survey should collect data beginning after February 1, 1998. This cut-off point was chosen because the article that linked the measles-mumps-rubella vaccine to autism and inflammatory bowel disease [48], and which caused grave doubts about vaccination among the public and some health professionals, was published on that date.

7. Languages:

The document must be written in English, Spanish, French, German, Italian, Dutch or Portuguese.

8. Geographic location:

The study must be performed in a developed country [49].

The main reason to exclude studies conducted in low-resource settings was that disease perceptions are different in areas where disease is less controlled, and this factor can significantly influence intentions to vaccinate [35,36].

9. Exclusion criteria:

Studies that measured the intention of HCW to vaccinate themselves, their family members or other HCW.
